# Incidence, prevalence and genetic determinants of neonatal diabetes mellitus: a systematic review and meta-analysis protocol

**DOI:** 10.1186/s13643-016-0369-3

**Published:** 2016-11-10

**Authors:** Jobert Richie N. Nansseu, Suzanne S. Ngo-Um, Eric V. Balti

**Affiliations:** 1 Faculty of Medicine and Biomedical Sciences of the University of Yaoundé I, PO Box 1364, Yaoundé, Cameroon; 2 Mother and Child Centre, Chantal Biya Foundation, Yaoundé, Cameroon; 3 National Obesity Centre, Yaoundé Central Hospital, Yaoundé, Cameroon; 4 Diabetes Research Centre, Vrije Universiteit Brussel-VUB, Laarbeeklaan 103, Brussels, Belgium; 5 Department of Clinical Chemistry and Radio-immunology, Universitair Ziekenhuis Brussel-UZ Brussel, Vrije Universiteit Brussel, Laarbeeklaan 101, Brussels, Belgium

**Keywords:** Epidemiology, Genetic: neonatal diabetes mellitus, Systematic review, Meta-analysis

## Abstract

**Background:**

In the absence of existing data, the present review intends to determine the incidence, prevalence and/or genetic determinants of neonatal diabetes mellitus (NDM), with expected contribution to disease characterization.

**Methods:**

We will include cross-sectional, cohort or case-control studies which have reported the incidence, prevalence and/or genetic determinants of NDM between January 01, 2000 and May 31, 2016, published in English or French languages and without any geographical limitation. PubMed and EMBASE will be extensively screened to identify potentially eligible studies, completed by manual search. Two authors will independently screen, select studies, extract data, and assess the risk of bias; disagreements will be resolved by consensus. Clinical heterogeneity will be investigated by examining the design and setting (including geographic region), procedure used for genetic testing, calculation of incidence or prevalence, and outcomes in each study. Studies found to be clinically homogeneous will be pooled together through a random effects meta-analysis. Statistical heterogeneity will be assessed using the chi-square test of homogeneity and quantified using the *I* ^2^ statistic. In case of substantial heterogeneity, subgroup analyses will be undertaken. Publication bias will be assessed with funnel plots, complemented with the use of Egger’s test of bias.

**Discussion:**

This systematic review and meta-analysis is expected to draw a clear picture of phenotypic and genotypic presentations of NDM in order to better understand the condition and adequately address challenges in respect with its management.

**Systematic review registration:**

PROSPERO CRD42016039765

**Electronic supplementary material:**

The online version of this article (doi:10.1186/s13643-016-0369-3) contains supplementary material, which is available to authorized users.

## Background

Neonatal diabetes mellitus (NDM) is a severe and monogenic form of diabetes mellitus (DM) which is characterized by the onset of insulin-requiring hyperglycaemia within the first months of life. NDM can be either transient or permanent. Transient NDM presents soon after birth and undergoes spontaneous remission during infancy; it may however relapse to a permanent form of DM in childhood or adolescence. Permanent NDM, which accounts for 40–50 % cases of NDM, is rather a form of DM that occurs during the first 6 months of life and does not go into remission, with patients often presenting with failure to thrive, vomiting, dehydration, poor feeding, hyperglycaemia and ketosis [1–7]. NDM has been estimated to occur in 1 over 20,000 to 500,000 live births [3, 8–11], but no clear estimate is available. The aetiology of NDM can be attributed to the missing or disturbed development of the pancreas, reduced pancreatic β cell mass, disturbed β cell function or early islet cell destruction [5].

There is strong evidence that a genetic diagnosis of NDM improves the treatment [7]. For instance, neonates suffering from NDM caused by a potassium channel gene mutation are very sensitive to sulfonylurea treatment; hence, their clinical management can be substantially improved by replacing insulin by oral agents [12]. However, traditional genetic testing for NDM will require accurate clinical information about the patient’s phenotype to allow selection of a small number of genes to test. Further, the selection of genes to be sequenced will also require a predictive list based on what has usually been registered so far. But to date, there is no existing data that has yet compiled the phenotypic and genotypic presentations of both forms of NDM, as well as factors determining their occurrence. We believe that a study addressing this issue is urgently needed, which will generate significant clinical impact in terms of a better characterization of the disease subtypes and consequential improvement in NDM management.

### Objectives

The present protocol is for a systematic review and meta-analysis to determine the following:

- The incidence of NDM
- The prevalence of NDM
- The genetic and/or epigenetic determinants of NDM

### Review questions

This review of studies published between January 01, 2000 and May 31, 2016, is designed to address the following questions:

- What is the global incidence of NDM?
- What is the global prevalence of NDM?
- What are the genetic and/or epigenetic determinants of NDM?

## Methods

The methodology used in the present review will comply with recommendations of the Centre for Reviews and Dissemination guidelines released in 2009 [13]. We will use the Preferred Reporting Items for Systematic Reviews and Meta-Analyses (PRISMA) guidelines as the template for reporting this review [14]. This protocol was written and presented according to the PRISMA-P 2015 guidelines [15] and registered with PROSPERO (ID = CRD42016039765). The PRISMA-P checklist can be found as Additional file 1.

### Eligibility criteria

Peer-reviewed original reports of observational studies (cross-sectional, cohort or case-control studies) on either the incidence, prevalence, genetic and/or epigenetic determinants of NDM will be systematically identified and appraised. These studies must have been conducted in human subjects, published between January 01, 2000 and May 31, 2016, and English or French languages without any geographical limitation. Intervention studies, letters, reviews, commentaries, editorials and reports of less than 10 cases will not be considered for this review. In case of duplicate reports, the most comprehensive and up-to-date version will be included. Table 1 gives a summary of inclusion and exclusion criteria.

**Table 1:** Inclusion and exclusion criteria

Inclusion criteria	Exclusion criteria
- Observational studies: cross-sectional, cohort or case-control studies	- Intervention studies, letters, reviews, commentaries, editorials and reports of less than 10 cases
- Report on either the incidence, prevalence, genetic and/or epigenetic determinants of NDM	- Duplicate reports (the most comprehensive and up-to-date version will be included)
- Conducted in human subjects	- Studies whose full data will not be accessible even after request from the authors
- Published between January 01, 2000 and May 31, 2016	
- Published in English or French languages without any geographical limitation	

*NDM* neonatal diabetes mellitus

### Information source

Publications will be identified by systematically searching two databases: PubMed/Medline and Excerpta Medica Database Guide (EMBASE). Literature search will be supplemented by screening bibliographies of identified articles and other pertinent review papers, conference proceedings and specialist journals (by visiting their websites).

### Search strategy and study selection

We will conduct a comprehensive and extensive search of the literature to identify all appropriate publications available from January 01, 2000 to May 31, 2016, and responding to our inclusion criteria. Table 2 represents the strategy that will be used for searching PubMed. Similarly, EMBASE will be searched using keywords related to epidemiology, incidence, prevalence, genetics or epigenetics and NDM.

**Table 2:** PubMed search strategy

Search	Query	Items found
#1	diabetes of the new born [Text Word] OR neonatal diabetes mellitus [MeSH Terms] OR neonatal diabetes mellitus [Text Word] OR diabetes mellitus in the neonate [Text Word] OR diabetes mellitus in the neonate [MeSH Terms] OR infancy diabetes mellitus [Text Word] OR permanent diabetes mellitus of infancy [Text Word] OR transient diabetes mellitus of infancy [Text Word] OR monogenic diabetes [Text Word]	10,070
#2	Epidemiology [Text Word] OR incidence [Text Word] OR prevalence [Text Word]	1,942,690
#3	Genetic factors [Text Word] OR risk factors [Text Word] OR ABCC8 OR KCNJ11 OR INS OR GCK OR PDX1 OR epigenetics [Text Word] OR gene expression regulation [Text Word]	1,584,221
#4	#1 AND #2	3860
#5	#1 AND #3	2587
#6	#4 OR #5	5019
#7	#6 Limits: from 2000/01/01 to 2016/05/31, and studies done in Humans	3433

After developing and piloting a screening guide to make sure that inclusion criteria are adhered to and consistently applied by all review authors, two authors (JRNN and EVB) will independently review all retrieved citations on the basis of titles and abstracts. Subsequently, for all records deemed relevant or potentially relevant, two authors (SSNU and JRNN) will independently assess the full-text articles for eligibility. The Cohen’s Kappa statistic will serve to measure agreement between review authors [16]. Discrepancies between review authors will be resolved by discussion and consensus; arbitration by a third author (EVB) will be sought whenever necessary.

### Collection of data

Data abstraction will be conducted by two independent researchers (SSNU and JRNN) using a preconceived and standardized abstraction form. Data will be pulled out from each study for the following variables: title, first author, year of publication, country(ies) of origin, geographical region, objective(s) and study design, study endpoint(s), assessment strategy, size of study population, age of participants (range), age at diagnosis, male/female ratio, subtype of NDM, history of consanguinity, incidence, prevalence, associated genes, risk factors and study conclusion(s). Disagreements between the two authors will be reconciled through discussion or arbitration by a third author (EVB).

Where only primary data (sample size/person time of follow-up and number of cases) will be provided, these parameters will be used to calculate the prevalence/incidence estimate. Where prevalence/incidence rates or relevant data for estimating them, or any other important information will not be available, the corresponding author of the study will be contacted at least twice to request the missing data.

### Quality assessment

Methodological quality of studies included in the meta-analyses will be assessed using the Newcastle-Ottawa scales pertaining to the design of each of the studies included [17]. Simultaneously, these instruments will be used to make an assessment of the risk of bias affecting study findings. All three researchers will independently assess the quality of studies included. The Cohen’s Kappa coefficient will serve to calculate degree of agreement between researchers and measure inter-rater agreement [16]. Additionally, a thorough description of missing data and dropouts for each included study will be provided, as well as the extent to which these missing data could have influenced study results.

### Data synthesis and analysis

Stata software v. 14 (Stata Corp, TX, USA) will serve for data analyses. We will present a flow diagram summarizing the process of identification of potentially eligible articles, with those that were subsequently excluded, and reasons for exclusion. A table will be used to present the main characteristics of included studies and the outcome of quality assessment of included studies. Summary statistics will include ranges, means (standard deviations) or medians (interquartile ranges) and frequencies (percentages) where appropriate.

Clinical heterogeneity will be investigated by examining the design and setting (including geographical region), procedure used for genetic testing, calculation of incidence or prevalence and outcomes in each study. Studies found to be clinically homogeneous will be pooled in the meta-analysis. The Cochrane’s Q statistic will be used to evaluate the presence of statistical heterogeneity (with a *p* < 0.10 being indicative of statistically significant heterogeneity) [18]. Besides, magnitude of statistical heterogeneity between studies will be assessed using the *I* ^2^ statistic (values of 25, 50 and 75 % will be considered to represent low, medium and high heterogeneity, respectively) [19]. Where substantial heterogeneity will be detected, a subgroup analysis will be performed using the following grouping variables: geographical region, assessment strategy, subtype of NDM, age at diagnosis and study methodological quality. If the included studies differ significantly in design, settings, outcome measures or otherwise, we will summarize the findings in a narrative format. Publication bias will be assessed with funnel plots, complemented with the use of Egger’s test of bias. Additionally, the trim-and-fill method will be applied to assess the impact of potential publication bias [20]. Statistically significant results will be set at a *p* value <0.05.

## Discussion

This systematic review and meta-analysis is expected to draw a clear picture of phenotypic and genotypic presentations of NDM in order to better understand the condition and adequately address challenges in respect with its management. This review does not require any ethical approval since it is based on published studies and not individual participant data. Its results will be published in a peer-reviewed journal and shared at relevant scientific conferences.

## Additional file

Additional file 1: PRISMA-P checklist. This document shows where the different items required in the PRISMA guidelines for protocols appear in the present document. (DOCX 24 kb)

## Abbreviations

DM: Diabetes mellitus
NDM: Neonatal diabetes mellitus
